# Advances in synthetic microbial ecosystems approach for studying ecological interactions and their influencing factors

**DOI:** 10.1016/j.engmic.2025.100205

**Published:** 2025-03-26

**Authors:** Wei Jiang, Sumeng Wang, Fei Gu, Xiaoya Yang, Qingsheng Qi, Quanfeng Liang

**Affiliations:** a State Key Laboratory of Microbial Technology, Shandong University, Qingdao 266237, China; b Research Center of Basic Medicine, Central Hospital Affiliated to Shandong First Medical University, Jinan 250013, China

**Keywords:** Synthetic microbial ecosystems, Ecological interactions

## Abstract

Investigating ecological interactions within microbial ecosystems is essential for enhancing our comprehension of key ecological issues, such as community stability, keystone species identification, and the manipulation of community structures. However, exploring these interactions proves challenging within complex natural ecosystems. With advances in synthetic biology, the design of synthetic microbial ecosystems has received increasing attention due to their reduced complexity and enhanced controllability. Various ecological relationships, including commensalism, amensalism, mutualism, competition, and predation have been established within synthetic ecosystems. These relationships are often context-dependent and shaped by physical and chemical environmental factors, as well as by interacting populations and surrounding species. This review consolidates current knowledge of synthetic microbial ecosystems and factors influencing their ecological dynamics. A deeper understanding of how these ecosystems function and respond to different variables will advance our understanding of microbial-community interactions.

## Graphical abstract

**Figure:**
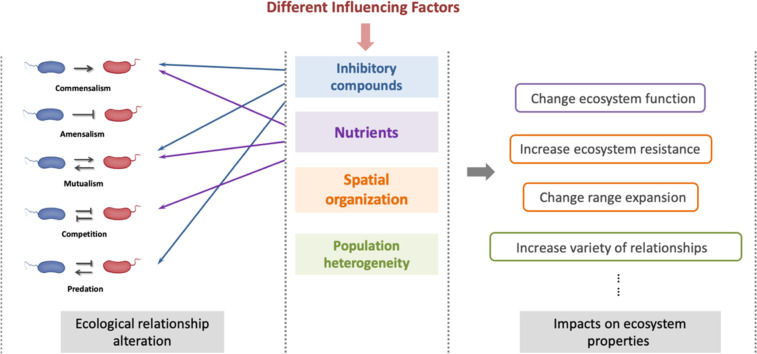

## 1 Introduction

A key focus of ecosystem biology is to investigate ecosystem characteristics at the community level, clarifying intra- and interspecific interactions [1,2]. Microbial systems are integral to maintaining the Earth's biosphere and human health, playing a pivotal role in diverse ecosystems [[3], [4], [5], [6]]. The tractability of microbes for laboratory experimentation makes them ideal subjects for ecosystem studies, leading to their widespread use in ecological research [[7], [8], [9]]. Microbial communities are characterized by considerable genetic and metabolic diversity, fostering various ecological interactions among populations [[10], [11], [12]]. Microbial ecosystems and sociomicrobiology are distinct but closely interrelated fields. Microbial ecosystems encompass communities of microorganisms and their physicochemical environments, focusing on material cycling, energy flow, and functional interactions within the microbial community and between microorganisms and their surroundings. Sociomicrobiology, on the other hand, investigates the collective behaviors and social strategies of microbes, particularly how they coordinate group actions through signaling molecules to facilitate social functions, including cooperation, competition, and cheating. The two fields are closely connected and complementary. Sociomicrobiology elucidates the molecular mechanisms underlying microbial behaviors, whereas microbial ecosystems examine how these behaviors shape community functions. Integrated application of both fields provides theoretical support for designing synthetic microbial communities from ecological and sociological perspectives. Understanding the evolutionary forces underlying ecological interactions is critical for deciphering community formation, function, and stability. However, the factors influencing these interactions remain largely unexplored.

In recent years, synthetic microbial ecology has attracted considerable attention [13]. Synthetic microbial ecology is a collective term for all rationally designed ecosystems where two or more defined microbial populations are designed in a well‐characterized manner and controlled environment. Building functional synthetic microbial ecosystems requires a systematic approach. The Design-Build-Test-Learn (DBTL) cycle proposed by Lawson et al. has become widely adopted and has been extensively applied in the field of synthetic biology [14]. The core principle is to optimize the construction and functionality of biological systems through iterative cycling. “Design” begins with defining functional objectives (e.g., bioproduction, bioremediation) and selecting interaction types (e.g., mutualism, competition) that align with ecological hypotheses or practical applications. The “Build” phase primary implements the rational bottom-up and evolutionary top-down approaches [15]. In the “Test” stage, computational modeling plays a pivotal role, employing dynamic frameworks such as the Lotka-Volterra equations or constraint-based metabolic tools to predict population trajectories [16,17]. In the final "Learn" phase, target functions are optimized via processes including high-throughput screening and directed evolution. This systematic approach accelerates the rational design of synthetic ecosystems and provides controllable models for elucidating complex interactions in natural communities [18]. Due to their reduced complexity and enhanced controllability, synthetic communities are often favored over natural ecosystems for investigating ecological theories, because managing the multifaceted variables of natural systems remains significantly challenging [13,19].

**Fig. 1:**
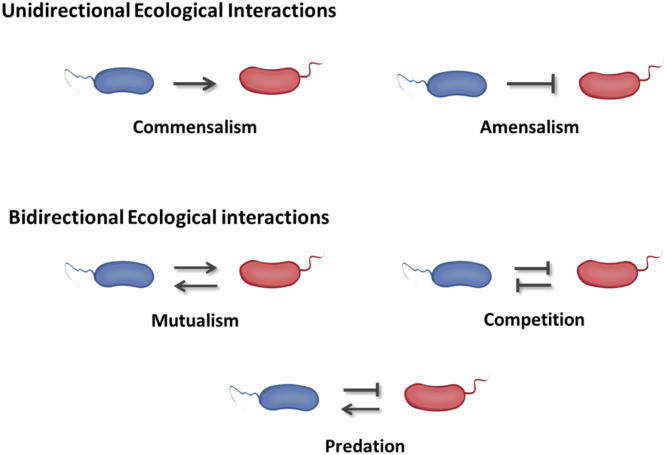
Different interaction modes within synthetic microbial ecosystems. Ecological relationships are categorized into unidirectional and bidirectional interaction types. Arrows indicate positive impacts on partner microorganisms, while flat arrows indicate negative impacts.

Synthetic microbial ecosystems are powerful tools for investigating evolutionary and ecological questions, including the generation and maintenance of biodiversity [20,21]. They provide a platform for examining the factors that shape microbial ecological interactions, including unidirectional (e.g., commensalism, amensalism) or bidirectional (e.g., mutualism, competition, predation) interactions (Fig. 1 and Table 1) [22,23]. Advances in synthetic biology have enabled the development of methodologies for designing robust synthetic communities [24]. The necessity of mathematical modeling has garnered increasing attention [[24], [25], [26], [27]]. Numerous methods and tools have been established to study microbial interactions and offer valuable insights into the roles of interacting species [28]. Investigating these interactions will deepen our understanding of complex and under-explored ecosystems.

**Table 1:** Synthetic microbial ecosystems with different interactions.

Relationships	Host	Mechanism	Ref.
Commensalism	*E. coli*-CHO	Acetaldehyde-induced regulation-controlled transcription of neomycin phosphotransferase	[45]
	*L. lactis*	Tetracycline resistance by nisin secretion	[46]
Amensalism	*E. coli*-CHO	AIR-controlled expression of the apoptosis-inducing human receptor-interacting protein	[45]
	*L. lactis*	Leveraging the bactericidal nature of nisin or lcnA	[46]
Mutualism	*S. cerevisiae*	Cross-feeding: essential amino acid- lysine and adenine	[50]
	*E. coli*	Cross-feeding: amino acid- isoleucine and leucine	[51]
	*E. coli*	Cross-feeding: amino acid-tryptophan and tyrosine	[52]
	*E. coli*	Cross-feeding: 14 essential amino acids	[53]
	*S. cerevisiae*	Cross-feeding: amino acids or nucleotide	[54]
	*Salmonella enterica ser. Typhimurium and E. coli*	Cross-feeding: methionine and metabolic waste	[55]
	*E. coli, Salmonella enterica serovar Typhimurium, B. thetaiotaomicron, and B. fragilis*	Cross-feeding: amino acids- l-methionine, l-histidine, l-tryptophan, and l-arginine	[56]
	*E. coli*	Cross-protection: ampicillin-resistance and Cm-resistance	[47]
	*E. coli*-CHO	Cross-protection: volatile acetaldehyde triggers β-lactamase (sBLA, hydrolyzes ampicillin) and NEO expression in CHO cells	[45]
	*L. lactis*	Cooperative nisin production, nisin induces tetracycline resistance in both strains	[46]
	*E. coli*	Cross-protection: QS system and toxin-antitoxin system	[19]
	*E. coli*	Cross-feeding and protection: intermediate products and *β*-lactamase enzyme	[57]
Predation	*E. coli*	QS system and antagonism protein CcdA/B	[58]
	*L. lactis*	Nisin and lcnA production	[46]
	*E. coli*-CHO	The predator (*E. coli*) inhibited the prey (mammalian CHO cells) by outgrowing the prey; the prey hydrolyzed ampicillin to promote predator survival	[45]
	*E. coli*-CHO	CHO-K1 engineered for constitutive sBLA expression to degrade ampicillin in the culture medium, enabling rapid growth of the parasitic *E. coli* in mammalian cell culture. *E. coli* exhausts nutrients and impairs mammalian cell growth	[45]
Competition	*L. lactis*	Antimicrobial features of nisin and lcnA	[46]
	*E. coli*	QS system and toxin-antitoxin system	[19]

Microbial ecosystems are highly context-dependent [29], with interactions shaped by both physical and chemical environmental parameters [[30], [31], [32]] and population-level heterogeneity [33,34]. These factors are considered pivotal determinants of ecosystem persistence and evolution [35]. However, limited understanding has hindered their broader application, an issue highlighted during the second wave of synthetic biology development [36]. Microbial ecosystems exhibit variable characteristics in different contexts, and interactions among populations are affected by various factors, posing significant challenges to ecologists working to gain profound insights into how ecosystems function and interact with different environments. Recent strides in synthetic biology suggest that synthetic ecosystems can provide critical insights into mechanisms underlying these dynamics [29,[37], [38], [39], [40], [41], [42]].

## 2 Synthetic microbial ecosystems exhibiting diverse interactions

### 2.1 Unidirectional ecological interactions: commensalism and amensalism

Commensalism and amensalism are unidirectional interactions in which one species affects another without experiencing reciprocal effects. A commensal effect is beneficial to the affected species, whereas an amensal effect is harmful [43]. Li et al. engineered *Escherichia coli* (*E. coli*) to establish metabolism and quorum sensing (QS) modules, enabling the rapid creation of microbial ecosystems with programmable ecological interactions [44]. Commensalism is facilitated through metabolite exchange, whereas amensalism is induced using a QS system and the toxin protein CcdB [44]. Weber et al. engineered a synthetic commensalistic *E. coli*-CHO ecosystem by modifying CHO cells to express acetaldehyde-induced neomycin phosphotransferase. Acetaldehyde production by *E. coli* enabled commensalistic CHO cell survival, whereas *E. coli* growth remained unaffected by separately cultured mammalian cells [45]. Weber et al. constructed an amensalism ecosystem in which CHO cells were engineered to perform AIR-controlled expression of an apoptosis-inducing human receptor-interacting protein, permitting survival only in the absence of *E. coli*-produced acetaldehyde [45]. Kong et al. utilized the modular nisin biosynthetic pathways in *Lactococcus lactis* (*L. lactis*) to develop a two-strain commensalistic consortium. One strain was engineered to secrete nisin, which triggered tetR expression in another strain and conferred tetracycline resistance [46]. They also constructed an amensalism consortium, in which one strain exerted a negative effect on another by exploiting the bactericidal properties of nisin and lactococcin A (lcnA) [46].

### 2.2 Bidirectional ecological interactions

#### 2.2.1 Mutualism

Mutualism refers to a reciprocal interaction in which both species benefit. Such interactions are vital for ecosystem stability and survival in challenging environments [38,47]. Mutualism primarily involves cross-feeding and cross-protection [38,[47], [48], [49]].

Shou et al. established a synthetic cross-feeding ecosystem using a pair of *Saccharomyces cerevisiae* (*S. cerevisiae*) yeast strains, each supplying an essential amino acid to the other [50]. Hosoda et al. engineered synthetic cross-feeding mutualism with two essential amino acid autotrophs in *E. coli* [51]. Kerner et al. constructed a cross-feeding mutualistic ecosystem capable of modulating growth rates and population composition by regulating the genes involved in the export or biosynthesis of cross-fed amino acids. By adjusting the metabolite exchange rate, the growth rate and population ratio within the synthetic ecosystem can be controlled [52]. Mee et al. developed a series of synthetic *E. coli* mutualistic communities to explore general properties that enable the metabolic exchange of amino acids. Fourteen essential amino acids were selected to create a range of cross-feeding ecosystems with increasing complexity. Their findings revealed that amino acids with higher biosynthetic costs fostered stronger cooperative interactions, and certain species exhibited keystone-like behaviors, significantly influencing community dynamics in more complex ecosystems [53]. Peng et al. developed a toolkit to manipulate two- and three-member consortia using different cross-feeding architectures of *S. cerevisiae* [54].

In addition to single-species mutualistic systems, multispecies interactions are common in natural microbial ecosystems. Harcombe demonstrated that cross-feeding cooperation could evolve in the laboratory through a two-species system involving *Salmonella enterica* ser. *Typhimurium* and mutant *E. coli* [55]. Ziesack et al. engineered interspecies amino acid cross-feeding mutualism using *E. coli, Salmonella enterica serovar* Typhimurium, *Bacteroides thetaiotaomicron*, and *Bacteroides fragilis* [56]. Their engineered cross-feeding mutualistic system promoted population evenness spanning environmental variation.

Cross-protection is another key mechanism for fostering cooperation in synthetic microbial ecosystems. This mechanism is exemplified by antibiotic degradation [[45], [46], [47]]. Yurtsev et al. established cross-protection mutualism in a multidrug environment using a pair of *E. coli* strains capable of antibiotic deactivation. In this system, growth-dilution cycles induced strong oscillations in the relative abundances of the two strains, revealing a periodic forcing effect that may be more characteristic of cross-protection than of cross-feeding mutualism [47]. Kong et al. developed a mutualistic system based on cooperative nisin production, in which two strains divide labor to synthesize nisin via a multistep process. Nisin induced tetracycline resistance in both strains, enabling them to survive in tetracycline-supplemented media [46].

In a previous study, we constructed a cross-protection synthetic mutualistic ecosystem. This system utilized orthogonal QS systems and a toxin-antitoxin module as respective communication and effect modules. Both strains were designed with negative feedback mechanisms to promote self-limitation, and positive feedback to enable population rescue. Antitoxin expression in each strain is triggered by signals produced by the other strain, resulting in mutual protection of both strains [19].

Cross-feeding and cross-protection can be integrated into a single system. For example, Li et al. designed a symbiotic ecosystem with two *E. coli* strains, one acting as a nutrient provider and the other as an antibiotic protector [57]. Compared with simpler mutualistic ecosystems, such as those based on either cross-feeding or cross-protection alone, these multifaceted mutualisms are more complex and offer a better reflection of natural ecosystem dynamics.

#### 2.2.2 Predation

Predation describes an ecological relationship in which a predator benefits at the expense of prey well-being. This is topologically similar to a combination of two unidirectional interactions, commensalism and amensalism, although in opposite orientations [46].

Balagadde et al. constructed a canonical synthetic predation system consisting of two *E. coli* strains that communicate *via* the QS system. The predator eliminates the prey by inducing toxin expression, whereas the prey protects the predator by triggering antitoxin expression [58]. Kong et al. have developed a predation system based on nisin and lcnA production. The prey, a nisin producer, possesses constitutive tetracycline resistance, whereas the predator, a lcnA producer, exhibits nisin-induced tetracycline resistance [46].

To enhance our understanding of biodiversity modulation, Song et al. analyzed the spatial and temporal dynamics of the predator-prey ecosystem they established. They observed that biodiversity decreased with increasing cellular motility when the segregation distance between the two populations was comparable to the length scale of chemically mediated interactions. This study provides clear criteria for predicting biodiversity changes through habitat partitioning and cellular motility in chemically mediated ecosystems [59].

#### 2.2.3 Competition

Competition involves an interaction in which the fitness of one species is reduced by the presence of another, resulting in a mutually detrimental relationship. Several synthetic competition ecosystems have been developed [40,46,60]. Kong et al. engineered a competition ecosystem using strains producing nisin and lcnA. Variants are created by modulating bacteriocin productivity, and different combinations of these variants yield distinct competitive outcomes [46]. This supports the idea that competition can be modulated by adjusting ecosystem parameters, such as relative interaction strengths.

In a previous study, we developed a competitive ecosystem using the same modules as the mutualism system described in Section 2.2.1. In this system, both strains possessed a positive feedback loop for self-rescue, and a negative feedback loop limiting population size. Toxin expression in each strain was induced by signals from the other strain [19].

## 3 Factors affecting ecological interactions in synthetic microbial ecosystems

**Fig. 2:**
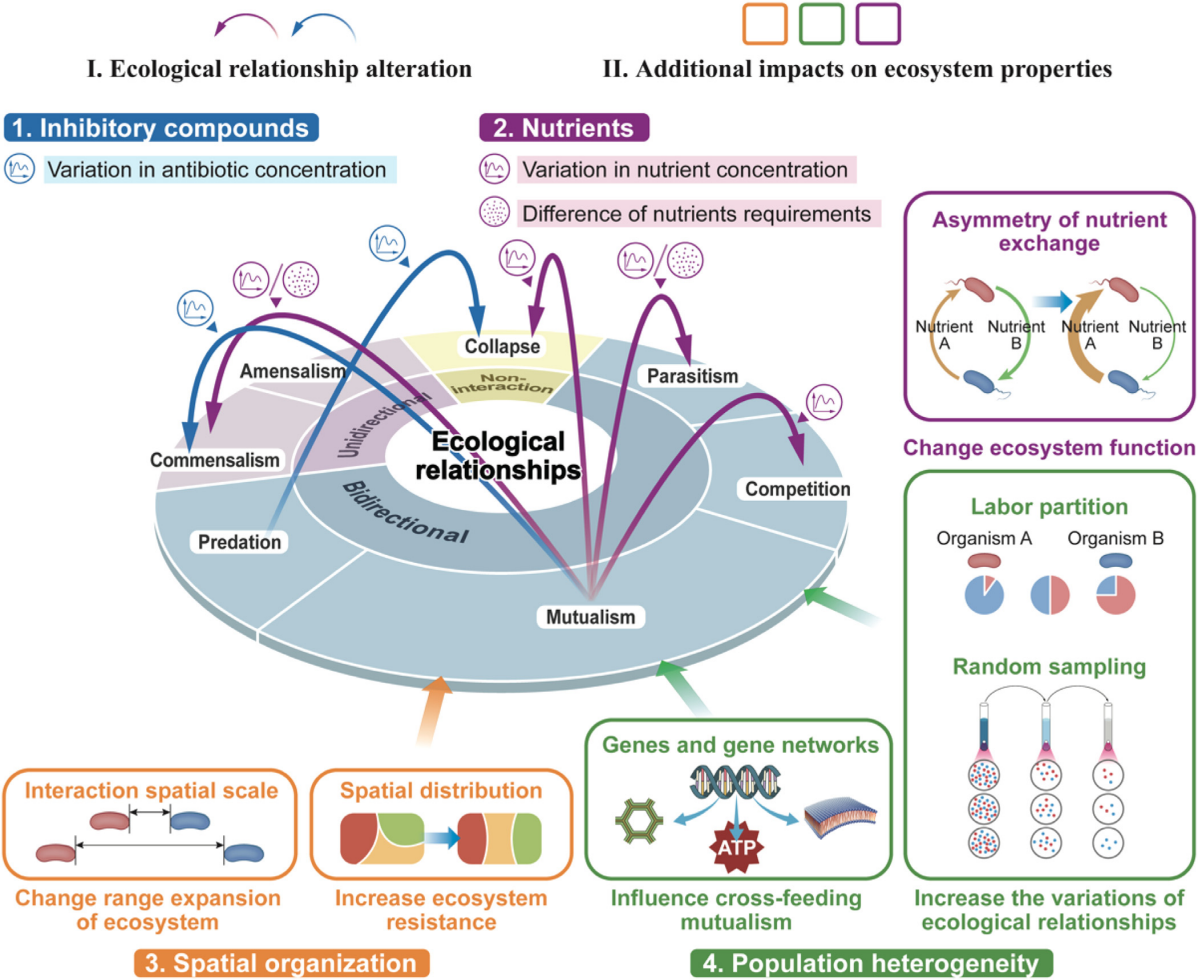
Various interactions and influencing factors within synthetic microbial ecosystems are depicted. Four key factors—Inhibitory compounds (blue), Nutrients (purple), Spatial organization (orange), and Population heterogeneity (green)—are illustrated alongside their respective effects on the ecosystem. These factors contribute to two primary outcomes: changes in ecological relationships (represented by curved arrows on the relationship map) and additional impacts on ecosystem properties (highlighted in boxes surrounding the map).

Synthetic microbial ecosystem function depends on delicate balances between several ecological and environmental factors. These factors, ranging from nutrient availability and microbial signaling to community composition and environmental conditions, are fundamental in shaping interactions between microbes. Many synthetic ecosystems are designed under the assumption that they will perform their intended functions regardless of environmental conditions. However, microbial ecological interactions are inherently influenced by cellular contexts in nature, which can lead to changes in ecosystem behavior. These interactions are highly variable and can be shaped by environmental parameters such as nutrients and pH levels [30,31]. These interactions also depend on the properties of the interacting cells and other microbial populations [41]. This section summarizes several specific factors that influence ecological relationships and offers insights into how these factors can be harnessed to optimize ecosystem design and functionality (Fig. 2 and Table 2).

**Table 2:** Factors affecting ecological interactions in synthetic microbial ecosystems.

Factors	Original relationship	Influencing factors	Influence consequences	Ref.
Inhibitory compounds	Cross-protection mutualism	Variation in antibiotic concentration	Ecological relationship alteration: Extinction, obligatory mutualism, commensalism for EG, facultative mutualism, and commensalism for ER	[37]
	Predation	Variation in antibiotic concentration	Ecological relationship alteration: predator dominance, prey-predator crossover, and ecosystem collapse	[29]
Nutrients	Cross-feeding mutualism	Variations in nutrient concentration	Ecological relationship alteration: obligatory mutualism, facultative mutualism, competition, parasitism, competitive exclusion, or collapse	[63]
	Mutualism	Differences in nutrient requirements	Ecological relationship alteration: commensalism or parasitism	[64]
	Cross-feeding mutualism	Asymmetry of nutrient exchange	Community function alteration	[30]
	Cross-feeding mutualism	Varied metabolite supplementations	Controls growth and population size in three-member co-cultures	[54]
Spatial organization	Competition	Interaction spatial scale	Change range expansion of ecosystem	[40]
	Cross-feeding and cross-protection mutualism	Spatial distribution	Increase in ecosystem resistance	[57]
Population heterogeneity	Bacteriocin-mediated cooperation and competition	Labor partition/random sampling	Increasing variations in ecological relationships	[41]
	Cross-feeding mutualism	Genes and gene networks	Community function alteration	[68]
	Cross-feeding mutualism	Promoter strength of metabolite exchange enzymes	Controls growth and population size in two-member co-cultures	[54]
	Cross-feeding mutualism	Initial cell densities	Controls growth and population size in three-member co-cultures	[54]

### 3.1 Inhibitory compounds

To elucidate the interplay between environmental conditions and ecosystems, inhibitory compounds, such as antibiotics, are frequently used as environmental factors in ecological succession studies [61]. Wright et al. showed that antibiotic inhibition contributes to biostability, promoting producer resistance to invasion at high abundance, and reducing their ability to invade at low abundance [62]. Kelsic et al. demonstrated that the opposing actions of antibiotic production and degradation facilitate coexistence, as indicated by simulations and analytical models [61]. Hu et al. designed, simulated, and constructed a synthetic cross-protection mutualistic ecosystem using two *E. coli* strains, ER and EG, which communicate via LuxI/R and RhlI/R QS signals. The signals produced by one strain activate a resistance gene in the other, enabling both strains to survive in antibiotic-containing environments. The authors observed that antibiotic concentrations influenced ecosystem population dynamics and classified them into five categories based on the ecological relationships between ER and EG: extinction, obligatory mutualism, commensalism for EG, facultative mutualism, and commensalism for ER. This study provides insights into how natural microbial ecosystems operate as models for understanding complex interactions among environmental factors [37].

Liu et al. constructed a predatory ecosystem using two *L. lactis* strains in which the prey mediated beneficial interactions by detoxifying antibiotics, and the predator inhibited the prey by producing lcnA. Their study showed that altering antibiotic concentrations in the environment led to ecosystem outcomes including predator dominance, prey-predator crossover, and ecosystem collapse. This highlights how the environmental dependence of microbial interactions can significantly enrich microbial community dynamics, leading to diverse community succession patterns under different initial conditions [29]. In a mutually beneficial bacterial community developed by Li et al., variations in antibiotic pressure can shift the relationship from parasitism to mutualism [57].

### 3.2 Nutrients

Nutrients are essential for organism survival, and their availability significantly impacts synthetic ecosystems. Several studies have investigated the effects of nutrients on microbial interactions. Hoek et al. investigated how changes in resource availability affect the interaction dynamics of cross-feeding mutualisms [38]. In this yeast-based cross-feeding system, one strain provided essential amino acids to its partner. Their findings showed that varying environmental amino acid concentrations led to six distinct interactions: obligatory mutualism, facultative mutualism, competition, parasitism, competitive exclusion, and population extinction [38]. Gore et al. found that glucose concentration can transform a snowdrift game into a prisoner's dilemma, highlighting the role of nutrient availability in modulating cooperative behaviors [63].

LaSarre et al. developed a mutualistic ecosystem involving fermentative *E. coli* and photoheterotrophic *Rhodopseudomonas palustris* (*R. palustris*), which cross-fed carbon (organic acids) and nitrogen (ammonium). To assess the influence of cross-feeding on ecosystem dynamics, the authors genetically engineered *R. palustris* to yield increased ammonium production. In parallel, *E. coli* was engineered to boost organic acid production, leading to culture acidification. As the organic acids transitioned from nutrients to inhibitors, the imbalance in nutrient exchange disrupted the species ratio, decreasing carbon conversion efficiency and demonstrating that disrupting nutrient symmetry can significantly alter ecosystem function [30].

Hammarlund et al. found that the propensity of nutrient addition to shift interactions from mutualism to either commensalism or parasitism depended on whether the nutrient was essential for the growth of one or both species [64]. Additionally, Peng et al. showed that varying metabolite supplementation influenced growth rates and population sizes in three-member co-cultures, further emphasizing the role of nutrient availability in shaping microbial interactions [54].

### 3.3 Spatial organization

Microorganisms in nature predominantly exist in complex communities, where spatial organization plays a pivotal role alongside functional interactions in shaping the ecological dynamics [65,66].

To investigate the effect of spatial interference on ecosystem organization during range expansion, Ozgen et al. constructed a two-strain *E. coli* competition system [40]. One strain exhibited contact-dependent short-range inhibition (SRI), while the other employed diffusion-based long-range inhibition (LRI). The SRI consortium includes a toxin-producing strain (SRI^+^) and a sensitive strain (SRI^−^). SRI^+^ inhibits the growth of SRI^−^ by directly injecting toxins (CdiA) *via* contact. In contrast, LRI inhibition occurs through an acyl-homoserine lactone (AHL)-inducible toxin gene. LRI^+^ secretes AHL, which diffuses across distance to induce toxin production and subsequent apoptosis in LRI^−^. In unidirectional interference, the extinction time of the toxin-sensitive species inversely correlated with the spatial interference scale, whereas bidirectional interference led to the emergence of distinct monoculture colonies, depending on initial conditions. These findings underscore the pivotal role of spatial scale in regulating the expansion dynamics of microbial ecosystems [40].

Li et al. engineered two *E. coli* strains—a nutrient provider and an antibiotic protector—to establish a mutually beneficial bacterial system for investigating spatial coordination [57]. Under antibiotic stress, bacterial distribution patterns shift, leading to different spatial distributions that confer a collective growth advantage that surpasses functional cooperation. This study highlights that, beyond functional mutualism, spatial organization serves as an additional mechanism of cooperative survival, enhancing resilience in natural microbial communities [57].

### 3.4 Population heterogeneity

In addition to external environmental factors, intrinsic characteristics of the populations involved in these relationships play critical roles in shaping synthetic communities. Liu et al. constructed *L. lactis* consortia to explore the impact of interaction variability on ecosystem succession and found that incorporating such variability into models improved the accuracy of bottom-up predictions. A cooperative consortium was formed through the production of lactococcal bacteriocins. For a consortium of bacteriocin-mediated cooperation and competition, altering labor partitions or random sampling can increase the variation in cooperation and drive the community to form distinct structures [41].

Other studies have investigated the effects of varying initial inoculum sizes and ratios on synthetic systems. These findings have revealed that the context-dependent nature of cellular interactions allows changes in initial inoculum to significantly influence the realization of ecological relationships, and overall community composition [29,54,57,67].

Building on prior work, LaSarre et al. employed random barcode transposon sequencing (RBTnSeq) to conduct a genome-wide search for *E. coli* genes influencing mutualistic interactions [30,68]. Several identified genes were linked to nitrogen sensing and assimilation, while others were involved in diverse cellular processes, including energy metabolism, cell wall formation, and membrane biogenesis. These results highlight the modulatory role of genes and gene networks in shaping cross-feeding mutualism, and provide deeper insights into the genetic underpinnings of microbial interactions. These findings also emphasize that microbial interactions are unpredictable. In a related study, Peng et al. demonstrated that the promoter strength of metabolite exchange enzymes directly governs growth and population size in two-member cocultures. This finding further highlights the crucial roles played by intrinsic characteristics of populations in shaping community formation [54].

## 4 Interactions between influencing factors in dynamic environments

Synthetic microbial ecosystems function within dynamic environments, where interacting factors may either synergistically or antagonistically affect ecosystem behavior. Understanding the interplay of factors influencing relationships within synthetic microbial consortia is critical to understand their behavior in dynamic environments. Studies have highlighted the complex feedback loops and interdependencies that drive the spatial patterns in microbial communities [69]. These factors include cell growth, movement, and chemical and physical environments, all of which are dynamically interconnected. For instance, cell growth and movement are influenced by the local chemical environment, which, in turn, is modulated by cellular metabolism and physical interactions. This interplay can lead to emergent properties, such as the formation of spatial patterns that influence community dynamics and function.

The stability and functionality of synthetic microbial consortia in dynamic environments are determined by the nonlinear interplays among metabolic dependencies, environmental perturbations, and spatial constraints. Metabolic cross-feeding, a key relationship-driven factor, establishes obligate interdependencies between species but also introduces fragility when resource gradients fluctuate [53,70]. For instance, syntrophic interactions optimized under steady-state conditions may fail under nutrient pulses due to mismatched growth kinetics [53]. Environmental factors such as pH and oxygen gradients further modulate these interactions by selectively enriching taxa with adaptive traits (e.g., acid tolerance or facultative anaerobiosis), thereby reshaping community topology [71].

Recent advances in integrating computational models have helped elucidate these dynamics. Genome-scale metabolic models (GEMs) coupled with agent-based simulations reveal how stochastic perturbations (e.g., phage attacks or antibiotic pulses) destabilize communities by disrupting the metabolic roles of keystone species [71,72]. Machine learning (ML) frameworks further predict resilience thresholds by correlating interspecies interaction networks with environmental stressors [70,72,73]. However, challenges remain in scaling these models to real-world scenarios, where multifactorial stressors induce emergent behaviors that were not observed in single-factor studies.

In dynamic environments, the interplay among these factors may lead to both predictable and unpredictable outcomes. For example, the introduction of engineered microorganisms into natural ecosystems may result in unintended consequences if the interactions between the synthetic consortia and the environment are not fully understood. It is thus crucial to consider the interplay between influential factors when designing synthetic consortia for real-world applications. This includes understanding how changes in one factor may affect other factors, and how these changes may influence the overall stability and functionality of a system.

## 5 Emerging trends in synthetic microbial ecosystems

Synthetic microbial ecosystems are exhibiting an emerging trend of multidomain integration. Artificial intelligence (AI) has become a pivotal tool for modeling complex community dynamics, optimizing metabolic networks, and accelerating the DBTL cycle. When integrated with multi-omics data, AI-driven platforms can be used to identify keystone species and metabolic bottlenecks. The convergence of ML and computational modeling represents a transformative paradigm for designing and predicting microbial community behaviors [73]. Conventional mechanistic models often fail to accurately represent the nonlinear dynamics of interspecies interactions, including metabolic cross-feeding, competition, and spatial heterogeneity [74]. ML techniques, such as deep learning and reinforcement learning, mitigate these limitations by extracting latent patterns from high-throughput omics data, enabling phenotype prediction from genotype variations without relying on predefined mechanistic assumptions [75]. For instance, ML-enhanced agent-based models have been employed to predict microbial interaction networks by analyzing self-organized spatiotemporal patterns even in poorly characterized communities [76]. Computational frameworks, such as GEMs, improve predictive accuracy by integrating multi-omics data to simulate community-level resource allocation and metabolic fluxes [77,78]. Hybrid approaches that integrate mechanistic models with data-driven ML can enhance both scalability and predictive robustness. Despite these significant advances, several challenges remain unresolved. ML models rely on large, high-quality datasets, the availability of which is limited in microbial ecology due to experimental variability. Interpretability also remains a major hurdle, as “black-box” ML models obscure biological insights. Future research should prioritize the integration of automated experimental platforms with ML pipelines to refine the design-predict-test cycle, ultimately advancing predictive biology for applications in precision medicine and sustainable agriculture.

CRISPR technology is a powerful emerging platform for microbial genome editing, enabling precise modification of target strains and optimization of microbial community functions. CRISPR-Cas systems, augmented by ML algorithms, offer species-specific editing capabilities to engineer microbial interactions (e.g., nitrogen-fixing bacteria) while minimizing off-target effects, thereby enhancing the stability of synthetic communities in heterogeneous environments, such as soil [79]. Microfluidic technology enables the simulation of complex microbial microenvironments and facilitates high-throughput screening and culturing. Microfluidic systems, particularly 3D-printed and paper-based devices, support high-throughput testing of synthetic consortia under precisely controlled spatiotemporal conditions, replicating real-world variability in nutrient availability and microbial competition [80].

Emerging hybrid systems, such as those integrating AI, CRISPR, and microfluidics, exemplify a transition toward autonomous, self-regulating microbial ecosystems with adaptive responses to environmental fluctuations. These innovations underscore the necessity for interdisciplinary approaches that integrate synthetic biology, systems modeling, and ecological engineering. Future research should prioritize scaling these technologies to enhance climate resilience and advance circular bioeconomy objectives, while ensuring the ethical deployment of engineered ecosystems.

## 6 Challenges and limitations

Synthetic microbial ecosystems hold great promise for various applications. However, their implementation is hindered by their inherent biological complexity and environmental variability. Accurate prediction of interspecies interactions in dynamic environments represents a major challenge because metabolic cross-feeding and competition generate nonlinear dynamics that defy conventional modeling. Another major obstacle is the scalability of synthetic communities and their functional redundancy. Although ML models can optimize community composition *in silico*, their predictions often overlook the effects of evolutionary drift and horizontal gene transfer in vivo. Metabolic conflicts also persist, particularly when engineered pathways compete for shared precursors and require extensive metabolic optimization. Additionally, ethical and biosafety concerns arise from the potential environmental release of synthetic consortia owing to our limited understanding of their interactions with native microbiomes.

Recent advances have encompassed dynamic gene circuits that enable real-time population control and 3D-printed microhabitats, facilitating spatial organization. However, the widespread adoption of these strategies has been hindered by their reliance on specialized equipment and the absence of standardized protocols. Overcoming these challenges necessitates the seamless integration of computational modeling, evolutionary stability strategies, and comprehensive risk assessment frameworks to effectively translate theoretical designs into practical applications.

## 7 Ethical and regulatory considerations

While synthetic microbial ecosystems serve as effective platforms for elucidating complex interactions within microbial communities, their design and application must consider ethical and regulatory considerations. The deployment of synthetic microbial communities in clinical, agricultural, and environmental settings necessitates comprehensive ethical evaluation and flexible regulatory frameworks.

For applications in human health, the design of synthetic communities must account for the diversity of the global human population to mitigate the risk of exacerbating health disparities. Prior to clinical implementation, rigorous screening of pathogens and virulence factors is required, and multicenter long-term studies are essential to evaluate potential risks, including the unintended proliferation of microbial consortia and horizontal transfer of antibiotic resistance genes.

Synthetic communities have the potential to disrupt ecological stability in both natural and agricultural settings. The ecological and ethical ramifications of unintended consequences of release of engineered microbes into natural ecosystems are significant concerns. These microbes can alter ecosystem dynamics, outcompete native species, and introduce unpredictable ecological perturbations. Concerns regarding microbial welfare have also been raised, although this remains a subject of ongoing debate.

With advances in this field, an integrated ethical and regulatory framework is essential to ensure that the development of synthetic microbial ecosystems promotes safety and societal and environmental benefits [81].

## 8 Discussion and concluding remarks

As synthetic biology progresses toward real-world applications, synthetic microbial ecosystems have emerged as a promising frontier owing to their enhanced functionality. The construction of these systems holds the potential to deepen our understanding of natural ecosystems in several key ways: 1) Validating Ecological Theories – Synthetic ecosystems act as "minimal models" to test hypotheses about natural communities; 2) Revealing Hidden Mechanisms – Simplified synthetic systems expose mechanisms that are often obscured in more complex natural environments, such as context-dependent interactions and evolutionary trade-offs; 3) Guiding Ecosystem Management – Insights from synthetic ecosystems can inform strategies for manipulating natural communities, such as in bioremediation and pathogen control.

Synthetic microbial ecosystems, characterized by modular divisions of labor and dynamic regulation, have demonstrated significant application potential in industrial production, environmental protection, and medical fields. In industrial biomanufacturing, microbial consortia designed using modular approaches significantly enhance product synthesis efficiency through metabolic division of labor. An exemplary case is the *Corynebacterium glutamicum*-*Pseudomonas putida* consortium, which produces γ-glutamylisopropylamide (2.8 g/L) and theanine (2.6 g/L) through arginine auxotrophy coupling and formamidase-mediated nitrogen exchange [82]. Synthetic microbial communities are used in bioremediation and pollutant degradation. For example, engineered microbial consortia have been shown to effectively degrade organic pollutants in soil and water [83]. Synthetic microbial communities are employed in medicine to investigate microbe-host interactions and develop novel probiotics and therapeutic strategies. For instance, studies employing synthetic microbial consortia to explore the relationship between gut microbiota and host health have provided new insights into disease treatment [84,85].

Microbial interactions are inherently variable and influenced by environmental conditions and variability inherent within interacting organisms. For example, bacteriocin production—a widespread antimicrobial strategy among microbes—is modulated by environmental factors including pH and ion concentration [34,86], as well as by the bacterial density of producers, as observed with nisin from *L. lactis* and subtilin from *Bacillus subtilis* [87,88]. Close coupling between microbes and their environment further complicates ecosystem dynamics. Microorganisms continuously absorb substrates and release metabolites, thereby modifying their surroundings, which in turn influences ecosystem behavior [89,90]. This microbe-environment coupling inherently complicates ecosystem behavior [91]. Thus, understanding context-dependence is essential for the design and description of synthetic microbial ecosystems.

When constructing synthetic ecosystems, it is crucial to account for the potential impact of environmental variables and emphasize context-dependence in microbial community design. A more profound understanding of how engineered ecosystems interact with their environments will be key to optimizing synthetic microbial ecosystems with desired functionalities, benefiting applications in both the health and environmental domains.

Most synthetic ecosystems currently consist of a limited number of species, restricting their resemblance to natural ecosystems and their practical applicability. To advance synthetic biology in real-world, complex environments, enhancing engineering capabilities for diverse, multispecies consortia is crucial. The next step in synthetic ecology should thus focus on developing ecosystems that increasingly mirror the natural environment. Models that simulate the inherent complexity of nature are pivotal for advancing our ability to explore the mechanisms and evolution of ecosystems.

## Declaration of Competing Interest

The authors declare that they have no known competing financial interests or personal relationships that could have appeared to influence the work reported in this paper.
